# Topical Tranexamic Acid Reduces Postoperative Blood Loss in Primary Total Hip and Knee Arthroplasty

**DOI:** 10.51894/001c.6942

**Published:** 2018-09-26

**Authors:** Corey M. Caruthers, Brett G. Brazier, Michael J. Blackmer, Sandra Raehtz, Gracia Etienne

**Affiliations:** 1 Orthopedic and Spine Specialists. York, PA; 2 College of Osteopathic Medicine, Michigan State University, East Lansing, MI; 3 Department of Orthopedic Surgery Residency Program, McLaren Greater Lansing, Lansing, MI

**Keywords:** allogenic blood transfusion, total hip arthroplasty, total knee arthroplasty, topical tranexamic acid

## Abstract

**CONTEXT:**

The purpose of this study was to assess the effectiveness of topical pre-closure application of tranexamic acid (TXA) to reduce postoperative blood loss and blood transfusion rates in primary total hip and knee arthroplasty (THA and TKA) in a private, high-volume orthopedic specialty hospital setting.

**METHODS:**

This was a retrospective study examining 140 consecutive patients undergoing primary hip or knee arthroplasty at the sample setting by a single surgeon. The first 70 patients did not receive topical TXA (2 gm./20ml.), the final 70 did receive topical TXA. We compared the postoperative hemoglobin levels of both sample subgroups at postoperative days 1, 2, and 3.

**RESULTS:**

Overall, the postoperative hemoglobin levels were significantly higher in the TXA group on postoperative days 1, 2, and 3 (p < 0.05). When patients who underwent THA (n = 70) were investigated separately, the hemoglobin levels were significantly higher on postoperative days 1, 2, and 3 in the group that received TXA. In the TKA group (n = 70), there was not a significantly higher hemoglobin level in patients who received TXA. There were no blood transfusions in the entire study cohort. Possibly due to the more restrictive transfusion criteria employed in this study, the total estimated prospective cost savings from use of TXA was calculated at about $116 per patient.

**CONCLUSIONS:**

Based on these results from a high volume orthopedic specialty hospital, pre-closure topical TXA application may prove effective in reducing postoperative blood loss for some patients but have a relatively small impact on cost outcomes.

## INTRODUCTION

The application of tranexamic acid (TXA) has become an emerging therapy proposed in the joint arthroplasty literature as surgeons have become increasingly aware of complications relating to allogenic blood transfusion (i.e., transfusion of blood from a donor).^1,2^ These include: transmission of infectious agents, postoperative infections and wound complications, transfusion reactions, intravascular coagulation, volume overload, renal failure, and short-term mortality.^1–6^ Furthermore, at a time when cost-effectiveness continues to be increasingly scrutinized, reducing blood transfusion and its associated expenses (e.g., laboratory acquisition/processing/storage costs, length of hospital stay, managing postoperative complications) continues to be an important topic.^1,5^

Traditionally, techniques for reducing blood loss in joint arthroplasty have included controlled hypotensive anesthesia, tourniquets with pressure-controlled pumps, autologous blood transfusion (i.e. collection and reinfusion of the patient’s own red blood cells), and local injections.^3,5^ Transfusion rates in primary hip and knee arthroplasty, however, continue to be elevated with some studies showing transfusion rates as high as 16 to 37%.^7^ Thus, efforts to reduce the use of allogenic blood transfusion have become a focus in orthopedics and the use of TXA has substantially increased over the last decade.^1,2^

TXA is a synthetic derivative of the amino acid lysine that carries out its effects through an antifibrinolytic pathway.^8–10^ TXA stabilizes formed clots and prevents the degradation of fibrin by reversibly inhibiting the lysine-binding site on plasminogen. This impairs plasminogen’s linkage with fibrin to become plasmin, which normally creates a fibrinolytic effect and dissolves clots. TXA was introduced in cardiac surgery more than 40 years ago, and is now increasingly used to control bleeding in cardiothoracic, trauma, obstetric and gynecological, gastrointestinal, urologic, and ear, nose, and throat surgeries.^8–10^

Due to the antifibrinolytic mechanism of action of TXA, there have been concerns expressed in the literature about the thrombogenic side effects of its administration, particularly with intravenous (IV) use of the drug.^10,11^ This continues to be the case even though there have been several meta-analyses completed showing no significant difference in the numbers of deep vein thrombosis (DVT) or pulmonary embolus (PE) events in patients receiving IV TXA.^10^

In fact, some studies have suggested fewer thromboembolic events with the use of TXA.^11^ Regardless, these theoretical risks have moved many surgeons away from the use of IV TXA.^1,2^ Regardless of the mixed results obtained to date, topical application of higher concentration TXA may be an alternative that provides similar benefits without concerns of possible systemic side effects.

### Purpose of Study

The aim of this study was to examine whether the pre-closure topical application of TXA helped to reduce postoperative blood loss and to evaluate whether topical TXA reduced the need for blood transfusion in a high-volume center. Additionally, a secondary goal of this study was to examine the validity of the debate on the effectiveness of topical TXA. A final goal of the study was to assess whether TXA is cost effective in primary total knee (TKA) or total hip arthroplasty (THA).

## METHODS

Before data collection, the Michigan State University intuitional review board certified the study as exempt from full review. A retrospective analysis was performed on 140 consecutive de-identified joint arthroplasty patients meeting the inclusion criteria. Inclusion criteria included all patients undergoing primary THA or TKA at a single institution by a single surgeon from April 2015 to September 2015. This institution was an orthopedic surgery specialty hospital where in 2017 there were 426 THA, 752 TKA and 152 unicompartmental (i.e., “partial”) knee arthroplasties completed for a total of 1,330 joint replacement surgeries.

Preoperative hemoglobin levels as well as postoperative hemoglobin levels for the first three postoperative days were required for inclusion into the study. Patient demographic data collected included age and BMI were also collected to assess any potential subgroup differences. The initial 70 analytic sample patients had not received topical TXA and the final 70 patients did receive topical TXA at the conclusion of surgery.

### Operative technique

All TKA surgeries were performed by a single surgeon. The surgeries involved either spinal or general anesthesia as well as administration of standard preoperative antibiotics. A midline incision with a medial parapatellar arthrotomy was used. A tourniquet was utilized in all cases and bleeding was managed with electrocautery. Implants included a cruciate retaining, fixed bearing total knee system. A standard Hemovac drain was placed prior to arthrotomy closure but not compressed until two hours postoperatively to remove any fluid or blood build-up in the joint. The group receiving TXA had it administered topically prior to closure of the arthrotomy at a standard dose of 2g/20mL.^8^

The same surgeon performed all of the THA surgeries with spinal or general anesthesia used for all surgeries. All THA procedures were completed through a direct anterior approach with use of an orthopedic table and press fit components were used for all patients. A Hemovac drain was placed at the conclusion of the case but not compressed until two hours postoperatively. In the group receiving TXA, it was topically administered prior to closure of the hip capsule using the same standard dose of 2g/20mL.^8^

Postoperatively, hemoglobin and hematocrit levels were drawn for three consecutive days. All patients received standard physical and occupational therapy protocols and received 24 hours of postoperative antibiotics. Enoxaparin and sequential compression devices were used for DVT prophylaxis. Discharge was typically on postoperative day 3. Preoperative and postoperative hemoglobin levels as well as patient demographics were retrieved retrospectively.

Statistical analyses were completed by the 2^nd^ (BGB) and 4^th^ author (SR) using GraphPad Prism version 7.00 for windows analytic software.^12^ All reported values represent the mean +/- the standard error of the mean (SEM). Gender comparison of hemoglobin levels were performed using one-way ANOVA with Fisher’s post-hoc test for paired subgroups. Cohorts were compared using two-way ANOVA with repeated measures using the Sidak multiple comparisons post-hoc test to examine differences between TXA treatments.

A two-way ANOVA was utilized to examine the influence that two different variables independently exerted on the single selected continuous outcome (i.e., postoperative hemoglobin levels). Additionally, Pearson’s correlation analyses were performed. A cut-off point of a p-value less than 0.05 was observed for all analytic procedures to indicate statistical significance.

## RESULTS

During the study, there were a total of 140 joint arthroplasty cases reviewed. There were no statistically significant differences found in age or BMI between patient subgroups that received TXA versus those that did not. (Table 1) This is important to note since a difference in these measures could have skewed subgroup outcome differences. Furthermore, using Pearson correlation analysis, there were no significant correlations found between age or BMI with postoperative hemoglobin levels (data not shown). While there was a statistically significant difference in hemoglobin levels between genders at each point measured, there was no difference in the percentage change of hemoglobin between males and females when compared to preoperative levels. Due to this, the analytic authors proceeded to group the genders for further analysis.

**Table:** Table 1 Patient Demographics between Groups. Values Represent the Mean +/- SEM. There Was No Statistical Significance between Groups in Regards to Demographics.

	**THA**		**TKA**		**THA + TKA**	
	**+TXA**	**-TXA**	**+TXA**	**-TXA**	**+TXA**	**-TXA**
N	34	36	36	34	70	70
BMI	33.24 ± 7.18	30.22 ± 6.68	33.36 ± 5.81	35.13 ± 7.74	33.3 ± 6.47	32.60 ± 7.58
Age	62.94 ± 9.38	66.14 ± 10.39	66.97 ± 7.56	66.82 ± 8.91	65.01 ± 8.67	66.47 ± 9.63
Male	13	18	4	9	17	27
Female	21	18	32	25	53	43
Transfusions	0	0	0	0	0	0

THA = Total Hip Arthroplasty

TKA = Total Knee Arthroplasty

The results of the 70 patients who underwent THA in the study are shown in Figure 1. Of these patients, 34 (49%) received topical TXA and 36 (51%) did not receive topical TXA. The mean hemoglobin of the patients who received TXA was 14.04 ± 1.12 g/dL preoperatively and 10.93 ± 0.79, 10.57 ± 0.72, and 10.72 ± 0.87 at postoperative days 1, 2, and 3 respectively. The mean hemoglobin of the group that did not receive TXA was 13.50 ± 1.22 g/dL preoperatively and 10.27 ± 1.11, 9.86 ± 1.12, and 9.87 ± 1.32 at postoperative days 1, 2, and 3 respectively. Statistical analysis demonstrated significantly higher hemoglobin levels in the group receiving topical TXA at postoperative days 1, 2, and 3 (p < 0.05) with no significant difference in preoperative hemoglobin levels.

**Figure:**
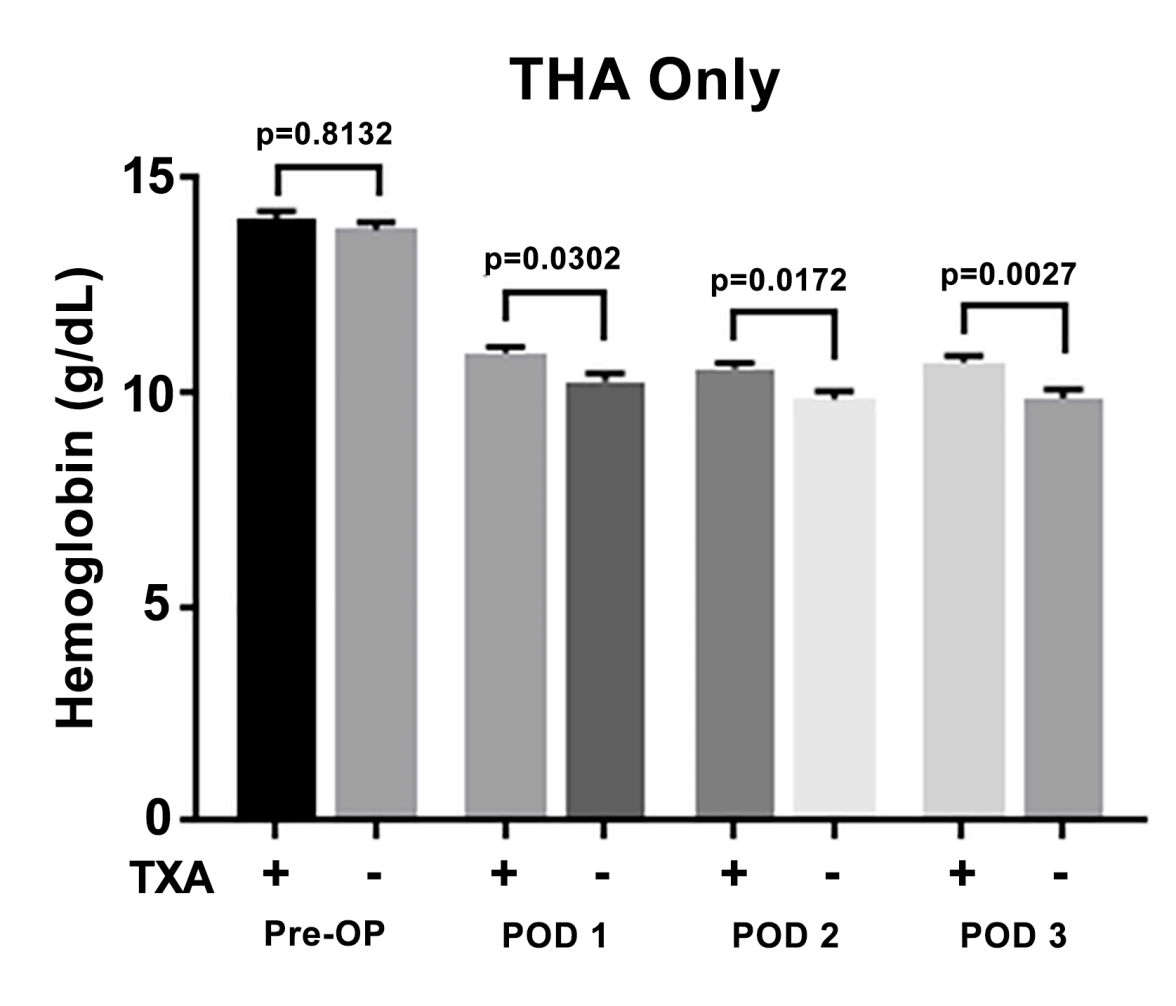
Figure 1 Total Hip Arthroplasty Cohort. Mean Hemoglobin Levels ± SEM (G/Dl) and Demographics of Patients Who Did (+) or Did Not (-) Receive Topical TXA. This Was Evaluated Pre-Operatively, On Post-Operative Day 1 (POD 1), POD 2, and POD 3. P-Value Considered Statistically Significant if < 0.05.

Data from the 70 patients who underwent TKA are shown in Figure 2. Of the 70 patients, 36 (51%) received topical TXA and 34 (49%) did not receive topical TXA. The mean hemoglobin of the patients who did receive TXA was 13.34 ± 1.03 g/dL preoperatively and 10.73 ± 1.03, 10.44 ± 1.22, and 10.36 ± 1.21 at postoperative days 1, 2, and 3 respectively. The mean hemoglobin of patients who did not receive TXA was 13.50 ± 1.22 g/dL preoperatively and 10.36 ± 1.34, 9.91 ± 1.46, and 9.70 ± 1.51 at postoperative days 1, 2, and 3 respectively. There was not a statistically significant difference between hemoglobin levels at postoperative days 1 through 3 and there was no significant difference in preoperative hemoglobin levels.

**Figure:**
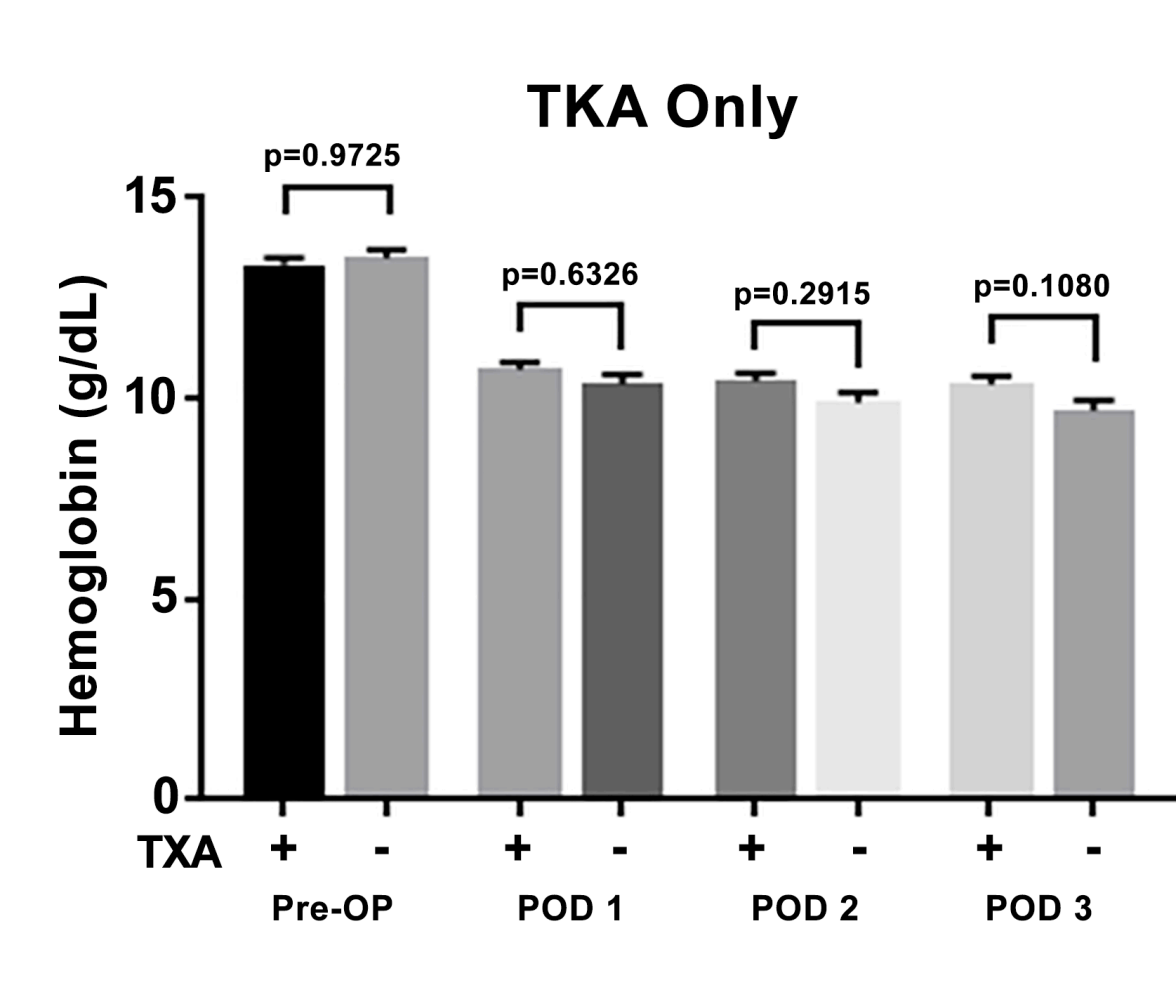
Figure 2 Total Knee Arthroplasty Cohort. Mean Hemoglobin Levels ± SEM (G/Dl) and Demographics of Patients Who Did (+) or Did Not (-) Receive Topical TXA. This Was Evaluated Pre-Operatively, On Post-Operative Day 1 (POD 1), POD 2, and POD 3. P-Value Considered Statistically Significant if < 0.05.

Figure 3 shows the analytic results of all patients (i.e., combined THA and TKA) comparing hemoglobin levels between those who did receive topical TXA with those who did not receive topical TXA. The mean hemoglobin of those patients who received TXA was 13.69 ± 1.12 g/dL preoperatively and 10.83 ± 0.92, 10.50 ± 1.01, and 10.53 ± 1.07 at postoperative days 1, 2, and 3 respectively. The mean hemoglobin of those patients who did not receive TXA was 13.66 ± 1.15 g/dL preoperatively and 10.31 ± 1.22, 9.89 ± 1.29, and 9.78 ± 1.41 at postoperative days 1, 2, and 3 respectively. Similar to the THA only group, the results of statistical analyses demonstrated significantly higher hemoglobin levels in the patients who received topical TXA at postoperative days 1 through 3 (p < 0.05).

**Figure:**
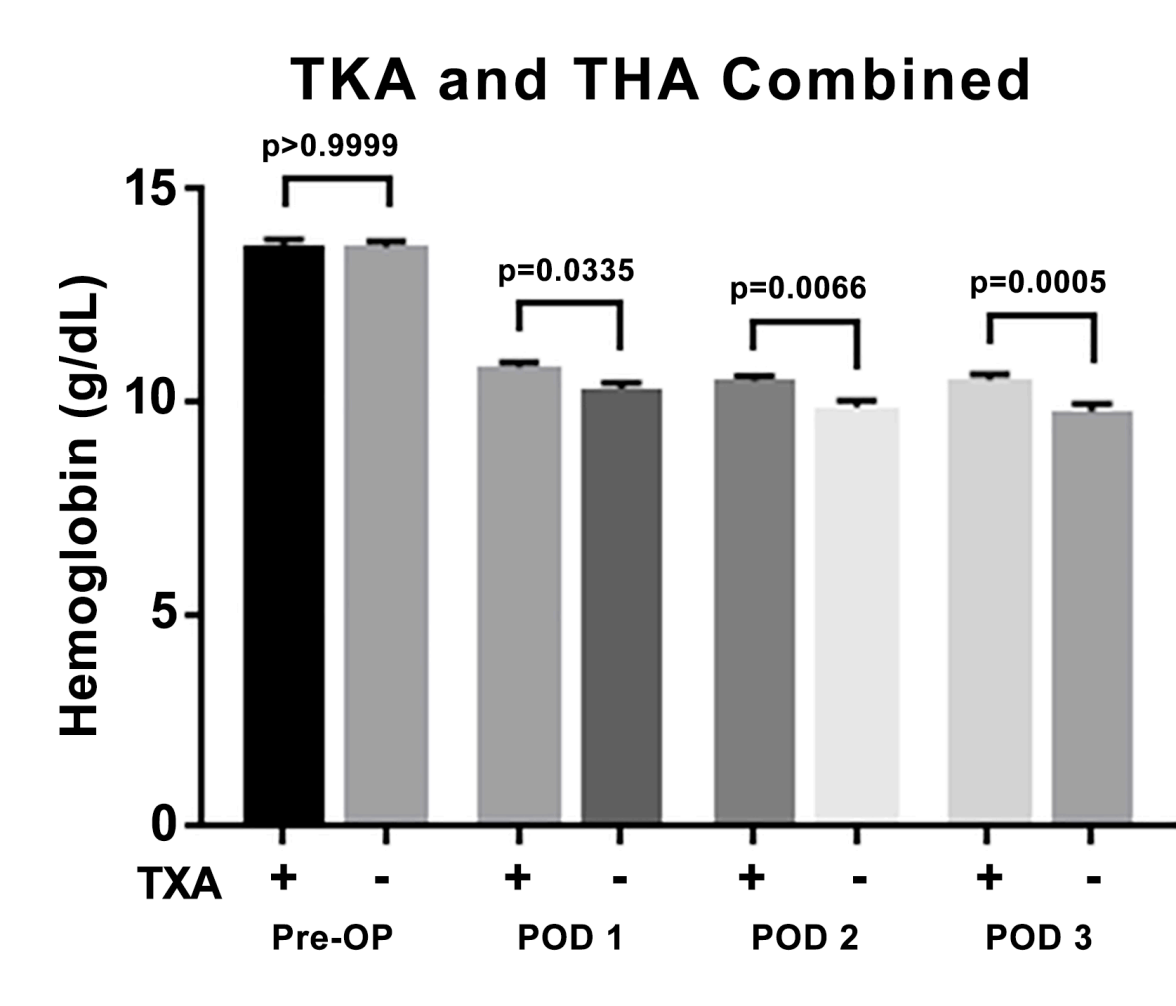
Figure 3 All patients (THA + TKA). Mean Hemoglobin Levels ± SEM (g/dL) and Demographics of Patients Who Did (+) or Did Not (-) Receive Topical TXA. This Was Evaluated Pre-Operatively, On Post-Operative Day 1 (POD 1), POD 2, and POD 3. P-Value Considered Statistically Significant if < 0.05.

There were only six (4.3%) sample patients in which postoperative hemoglobin levels ever dropped below 8 g/dL and none of them required blood transfusion. Of these patients, four (67%) were in the THA subgroup and two (33%) in the TKA subgroup. None of these patients whose hemoglobin levels dropped below 8 g/dL had received topical TXA.

## DISCUSSION

These results demonstrate that topical TXA can be effective at reducing postoperative decreases in hemoglobin levels after either primary THA or TKA. Overall, postoperative hemoglobin levels were significantly higher in the cohort of patients that received TXA compared to those that did not at postoperative days 1, 2, and 3. The effect of TXA appears to have been more pronounced in the THA subgroup, as the postoperative hemoglobin was significantly higher at all three postoperative days compared to the TKA subgroup, which did not have statistically significantly higher hemoglobin levels on any of the postoperative days.

In other settings, TXA has already been shown to be effective both topically and intravenously in multiple studies for both THA and TKA for reducing perioperative blood loss.^1,9,13–18^ In this study, the effect of topical TXA appears to be more pronounced in the THA subgroup. The reason for this is unclear but may be due to increased operative time, differences in intravenous fluid hydration, or the more extensive dissection generally required for the THA procedure.

Pursuant to institutional criteria, there were no patients in this study who required an allogenic blood transfusion. This 0% rate is significantly lower than the literature reported rates for blood transfusion for primary THA and TKA, which have been listed anywhere from 11 to 67%.^18–20^ Our intuitional transfusion guidelines matched the American Association of Blood Banks recommendations which comprised a restrictive transfusion strategy for patients with hemoglobin less than or equal to 8 g/dL and associated symptoms consistent with blood loss anemia.^2^

Other studies have also advocated for more restrictive transfusion criteria, finding no benefit for transfusion in patients with a hemoglobin level >8 g/dL regardless of cardiovascular risk.^20–22^ The authors’ adherence to the less stringent guidelines in this study would likely have led to transfusion of six patients in which the hemoglobin levels dropped below 8 g/dL but had not received topical TXA.

In 2013, Tuttle et al. performed a cost-benefit analysis of topical TXA in primary THA and TKA.^23^ In the study, they estimated the cost for transfusing one unit of packed red blood cells (PRBC) to be $787 and the cost of 1 g of TXA to be $58. They found a reduced transfusion rate from 17.5% to 5.5% after use of topical TXA and number of units transfused per patient dropped from 0.286 to 0.106 leading to a cost savings of $8373 per 100 patients treated or $83.73 per patient. Of note, the transfusion criteria observed in this 2013 study was a hemoglobin level of less than 8 g/dL or symptomatic anemia.^23^

If a similar cost-benefit analysis was performed using our study data, using the same transfusion criteria of hemoglobin < 8 g/dL (which would have led to a transfusion of six patients in the cohort who had not received topical TXA). This means that six (8.57%) out of 70 patients who had not received TXA would have been transfused and 0 (0%) of 70 non-TXA patients would have been transfused. Cost estimates based on a per patient transfusion of two units PRBC and with the reduction of 8.57% of allogenic units transfused ((787.37*8.57) x 2 units PRBC) totals $13,489.18.

Factoring in the cost of TXA and subtracting 2 grams used per patient for every 100 patients ($13,489.18 - $11,600.00), the cost savings would be $1,889.00 (per 100 patients treated) or $18.89 per patient. Assuming a transfusion of 1 unit of PRBC per patient, ((787.37*8.57) - 11600), the cost savings would have been $4,852.00 (per 100 patients treated) or $48.52 per patient. However, the use of the more restrictive transfusion criteria observed during this study period led to no patients being transfused and thus the total cost savings was $11,600.00 per 100 patients or $116 per patient.

These results suggest that the use of TXA may not be as cost beneficial if a restrictive blood transfusion criteria is used. If less stringent transfusion criteria had been utilized, however, the cost of topical TXA would partially offset secondary transfusion costs. Furthermore, a greater benefit of TXA use may be realized at institutions that have higher transfusion rates. The main goal from TXA use for most surgeons is to ultimately decrease the number of blood transfusions.^4–6^ However, the results of this study indicate that topical TXA applications had not reduced the number of transfusion events for this single high-volume arthroplasty surgeon.

### Study Limitations

We acknowledge several limitations to this study. First, all surgeries were performed at a high-volume orthopedic specialty hospital by one fellowship-trained arthroplasty surgeon. Because of this, there were shorter operative times and lower blood loss than would be expected for a lower volume surgeon and center and may account for the zero-transfusion rate that we found. This fact could limit the generalizability of our results to other settings. These results may not be representative of the significant number of TKAs performed at lower-volume hospitals typically associated with higher transfusion rates.^24^ However, the use of TXA may actually still be more beneficial for lower-volume orthopedic surgeons with longer operative times and higher blood loss.

Second, the study sample was comprised of a relatively smaller number of THA and TKA patients in Pennsylvania. In addition, there is the possibility for numerous unmeasured confounding influences on these results (e.g., variability in IV fluid administration and drain output). Finally, these were retrospective analyses subject to possible inherent weaknesses compared to prospective, randomized, and blinded data sources.

## CONCLUSIONS

Based on these results, pre-closure topical TXA application may prove effective in reducing postoperative blood loss but have less impact on transfusion rates or cost outcomes. Standardized doses of topical TXA were particularly shown to be effective in reducing perioperative blood loss in THA surgeries. These findings, however, failed to indicate any sizable cost savings derived from TXA use. Future studies are needed to analyze outcomes associated with topical TXA in varied orthopedic contexts such as high versus low volume joint arthroplasty settings, TXA use in revision cases, bilateral TKA procedures, etc.

### Conflict of Interest

The authors declare no conflict of interest.
